# A Comparative Analysis of the Response of GNSS Receivers under Vertical and Horizontal L1/E1 Chirp Jamming

**DOI:** 10.3390/s21041446

**Published:** 2021-02-19

**Authors:** Polona Pavlovčič-Prešeren, Franc Dimc, Matej Bažec

**Affiliations:** 1 Faculty of Civil and Geodetic Engineering, University of Ljubljana, Jamova Cesta 2, SI-1000 Ljubljana, Slovenia; 2 Faculty of Maritime Studies and Transport, University of Ljubljana, Cesta Pomorščakov 4, SI-6320 Portorož, Slovenia; franc.dimc@fpp.uni-lj.si (F.D.); matej.bazec@fpp.uni-lj.si (M.B.)

**Keywords:** intentional interference, geodetic and low-cost GNSS receivers, vertical/horizontal jamming profile, L1/E1 chirp jammer

## Abstract

Jamming is becoming a serious threat to various users of global navigation satellite systems (GNSS). Therefore, live monitoring tests are required to estimate the sensitivity range of GNSS receivers under jamming. This study analyses the response of some mass-market and professional-grade receivers to intentional interferences based on different 3D jammer positions. First, the vertical jamming was investigated, followed by a horizontal experiment where the receivers were placed at three locations while the jammer was moving within a triangular area. The aim was to determine a fingerprint of the influence of the L1/E1 chirp jammer on receivers used in the research. The results show that low-cost receivers are much more susceptible to interference, while the latest generation of GNSS geodetic receivers are much more resilient. It is encouraging that positioning in the presence of jamming could be achieved on a larger scale, especially by using professional receivers. An attempt to position the jammer will be left for trials when a more frequency stable device is applied.

## 1. Introduction

Today, awareness of the risk of using global navigation satellite systems (GNSS) in our daily lives and in any public or private infrastructure, known as “jamming”, is very important. In GNSS positioning, intentional jamming is considered the most disruptive event that drastically affects the reliability and quality of positioning or, in the worst case, makes positioning impossible [1,2]. Receiver manufacturers are well aware of this; hence, they invest a lot of effort in anti-interference features. However, under certain circumstances, GNSS equipment still proves to be susceptible to intentional interference [3]. Improving the integrity and security of GNSS signals is an important issue that should be considered to achieve continuous and uninterrupted positioning. Jamming transmitters in cars alone already pose a major threat to the security and operation of infrastructure. As a wide range of infrastructure around the world—including industry, broadcasting and telecommunications, automotive, smart cities, internet of things, aerospace and defence—are directly dependent on GNSS; all benefit from this accurate and trusted service to support their operations. GNSS are so vulnerable for the following reasons: (a) the signal specifications are open to anyone, see [4,5,6,7]; (b) no signal protection systems have been implemented, except for the encrypted GNSS services such as the global positioning system (GPS) P(Y) and M-code, GLONASS P-code, Galileo’s public regulated service (PRS), and BeiDou’s military service [8]; (c) an extremely weak signal is below the thermal noise of the receiver, i.e., −111 dBm for the GPS coarse acquisition (C/A) code signal [9]; (d) the initial transmission power of the satellite signal of 30 W is reduced by $10^{16}$ times at the receiver [10]; (e) jamming and spoofing devices are readily available on the market, although their use is strictly prohibited [11].

Jammers can be used in various scenarios; for example, they can be used in a static or kinematic mode, and they can be located on various vehicles, i.e., in cars or on the surface of aircraft. In the context of jamming, the driving motivation for the research presented in this paper was to perform some further experiments and to analyse the results in order to better understand some key factors that influence the quality of positioning under different locations of a jammer as well as under different jamming scenarios. Having studied the knowledge of the response of different types of geodetic receivers [2], the researchers extended this study to the problem of the receiver response under the vertical and horizontal jamming profile. The basic research question was, “How do GNSS receivers of the same type react to vertical jamming when the jammer is below or above the horizon of the GNSS receivers?” and secondly, “What is the quality of GNSS positioning while horizontal jamming?” More specifically, the research focused on two different issues: (a) the effects of the vertical jammer profile and (b) the ability to determine the presence of a jammer horizontally by using a simple network of GNSS receivers.

The results of the vertical jamming profile are important in connection with the establishment and stabilisation of GNSS stations, where the receivers can be mounted on pillars or higher on buildings [12]. If the jammer on the ground no longer interferes with the higher receivers on buildings, the suitability of such stabilisation is justified. Unfortunately, this raises another issue of the stability of buildings, which must be solved through a deformation analysis in order to obtain a good time series for geodynamic modelling or for maintaining the coordinate system. Furthermore, the problem of the vertical jamming profile also relates to unmanned aerial vehicles (UAV) equipped with GNSS-RTK (real-time kinematic), which allow the process of georeferencing by avoiding the determination of ground control points to be simplified [13].

The analysis of jamming can be difficult, because in various situations, jammers can only eliminate a part of the visible satellites, meaning that the position is available but the accuracy decreases. In this situation, warnings of drastic changes in receiver-satellite geometry between real and predicted conditions should take precedence. Therefore, in this study, the researchers also investigated carrier-to-noise-zero (C/N_0_) values. To estimate the positioning quality in geodetic applications, it is best to rely on the combined C/N_0_ values and the number of available satellites [14,15]. Unfortunately, the dilution of the precision factors (DOPs) cannot be relied upon, since a higher DOP does not always mean that jamming is present; however, it can occur due to obstacles along the line of sight, especially in kinematic positioning.

### 1.1. Overview

In recent years, several jamming detectors have been proposed that use different signal characteristics to recognise interference. The simplest and most commonly used device is an energy detector, which can be used in both the time and frequency domain. Balaei et al. presented a frequency domain detector for the identification of narrowband continuous wave interference signals [16]. Several measures were introduced for the detection of interference, namely estimation of the correlator output power, standard deviation of the correlator output power, carrier-phase fluctuation, and the gain of automatically adjustable controller between the analogue section of the front-end and the analog-to-digital converter [17]. In the field of signal analysis, signal-to-noise ratio (SNR) or carrier-to-noise density (C/N_0_) are often used for the detection of signal quality. Such detectors do not require access to the internal signal processing in the GNSS chipset, but they can detect any kind of signal interference. Unfortunately, they cannot distinguish between increased interference power and decreased GNSS signal power. This is the reason why C/N_0_ detectors can only be effective at fixed locations without random signal attenuation caused by buildings. In addition, interference from a combined inertial navigation system and GNSS sensors, namely GNSS/INS sensors, can be detected [18,19,20,21]. Effective interference detection is based on a network of multiple antennas that can exploit the spatial characteristics of the signals received from satellites and from a jammer and distinguish whether the signals came from different directions. A detector that exploits the spatial properties of a single moving antenna was introduced in [22]. As a countermeasure to jamming attacks, adaptive beam-forming techniques can be used for spatial filtering of a jamming signal. The advantage of multiple antenna arrays is that they can detect the jammer and are also able to mitigate the effects of interference by using beam-forming and nulling techniques [23].

The authors demonstrated that jamming can be easily detected [24] and localised [25]; however, practical experience shows the opposite, especially in cases where a jammer is in its kinematic mode. Simple crowdsourcing methods involving reporting signal losses [26] up to more sophisticated analyses of signal power or observed C/N_0_ values [27] are available to study the effect of interfering signals. The advantage of C/N_0_-based detectors is that they recognise any kind of signal interference without any additional requirements; however, at the same time, there is a serious disadvantage because C/N_0_ estimates decrease both in the case of interference of the GNSS receiver and that of attenuation of the desired GNSS signal by buildings or other obstacles. Therefore, C/N_0_ detectors are only suitable at fixed positions at roadsides or on critical infrastructure, whereas in mobile scenarios in urban environments, their usefulness is questionable.

When interference occurs, there are three ways to geo-locate it, namely by (a) the received signal strength or strength difference (RSS/RSSD), (b) the signal arrival angle or direction (AOA/DOA/RSSD), and (c) the signal arrival time or frequency difference (TDOA/FDOA) [28]. However, the results from all three methods depend to a large extent on different types of environments, for example, an open space or more complex suburban and urban environments. Faria et al. investigated jamming in different types of environments and in different propagation models based on the jamming-to-signal ratio (J/S) for a wide range of EIRP (effective isotropically radiated power) jamming signals at different distances in different environments, with the aim of estimating a safe zone for the operation of GNSS (GPS)-based systems [29]. Their study was based on the determination of the horizontal safety zone for jamming, where the height of the jammer was set at 60 m and the height of the GPS receiver was set at 2 m.

Much effort has been put into the development of products that allow the transmission of warnings or other functions to inform users of any changes detected between the noise and the jamming signal. In November 2019, Netradar joined the European Space Agency’s Business Incubation Centres (ESA BICs) to develop a solution based on GNSS performance data from mobile phones via the Netradar app. For the aviation industry, an innovative way to detect GNSS jamming incidents on air traffic data based on automatic dependent surveillance-broadcast (ADS-B) data from open-sky receivers was proposed in [30]. A more sophisticated approach that goes beyond warnings and tries to overcome jamming is based on the construction of anti-jamming receivers. A report on specially designed receivers with an anti-jamming-mechanism was presented by Felski [31]; however, unfortunately such receivers are not available to everyone. The author mentions two CHRONOS products, namely CTL3520 and CTL3510 GPS jammer detectors, which can recognise the presence of jammers in the L1 band. However, the average GNSS user is not able to locate the interfering transmitter.

The results of a previous study conducted at the University of Ljubljana showed that the response of receivers from different manufacturers and generations is unique for each type of receiver tested [2]. The aim of the present research is to determine the response of several receivers of the same type located in different positions to the same interfering signal. Evaluations of the quality of detection, tracking processes, and the possibilities of acquiring signals from GPS, GLONASS, Galileo, or BeiDou were performed in order to obtain a fingerprint of the influence of the L1/E1 chirp jammer on each individual GNSS receiver used in the experiment.

### 1.2. Previous Research

As GNSS jamming can be harmful to public, governmental, or commercial sectors, several studies have been carried out in the past. Different types of jammers and their characteristics have been investigated in [32], with special emphasis on multi-frequency broadband jammers, which are able to interfere with up to three frequencies simultaneously. Since jamming effects were analysed outside vehicles [32], a strong attenuation of metal structure was observed, while through-window directions were favoured. In [33], a GNSS localisation of jammers was proposed based on a vehicle ad hoc network within an accuracy of less than 40 metres to determine the origin of the jammers. Field tests for this study were carried out in the GATE Berchtesgaden test environment where eight ground transmitters around the test area played the role of Galileo satellites. AOA, TDOA, and differential-received-signal strength (DRSS) were used to geolocate the jammers. In [34,35], the localisation of jammers was performed with low-cost GPS receivers and smartphones that provided C/N_0_ measurements. In [16], C/N_0_ estimates for each satellite were used to detect a continuous wave (CW) interference signal. The research on the same basis, namely the approximation of the C/N_0_ estimate as a Gaussian to obtain a further decision threshold for each satellite and elevation, was performed in [36]. In this study, the authors have described a method for estimating C/N_0_ values that avoids the need to access the internal signal processing in the GNSS chipset.

In several outdoor jamming experiments [2,33,37,38,39,40], the authors limited themselves to horizontal jamming scenarios by specifying different distances of the jammers depending on the GNSS receiver. In addition, they investigated the jamming performance in environments with various obstacles, i.e., in suburban and urban areas [29], in most cases considering horizontal jamming scenarios. A deliberate jamming attack on GNSS receivers mounted on an aerial vehicle was presented in [41], in which the authors proposed a two-stage interference suppression scheme based on antenna arrays that could detect and mitigate jamming and spoofing. However, based on the authors’ knowledge, no experiments on jamming with multiple geodetic GNSS receivers under a vertical jamming profile, wherein the same kind of geodetic and mass-market receivers were placed simultaneously below and above the receiver’s horizon, have been performed to date. To the contrary, several studies analysed the effects of various jammers at different horizontal distances and different propagation models [29,42,43].

### 1.3. Paper Focus and Outline

Within the research area, the driving motivation for this study was to perform some further experiments under real jamming conditions and to analyse the results to get a better understanding of the performance of GNSS receivers under vertical and horizontal jamming profiles. The focus of this research is on the vulnerability of different types of GNSS receivers under two scenarios of jamming set up by radio frequency interference (RFI) for the GNSS L1/E1 spectrum. In order to achieve the goal, the authors conducted outdoor tests. First, the effects of static and kinematic jamming signals at the positions where the jammer was located below and above the receiver horizon were estimated. Secondly, the effects of kinematic horizontal jamming signals at the positions where the distances between the jammer and the GNSS receivers varied were estimated and tachymetrically determined within the jamming session. The key to this research is to gain experience by establishing a network of different types of GNSS receivers that could be used as C/N_0_ jamming detectors.

The remainder of this document is structured as follows: Section 2 describes the setup and measurement campaigns, which is followed by a description of the experiments. Section 3 describes GNSS observation models for jamming detection, followed by a description of data processing and the procedure for evaluating the results from the experiments (Section 4). Section 5 contains results and data analyses, including a discussion. Finally, Section 6 presents a summary of the most important results and a description of further ideas for experiments.

## 2. Materials and Methods

### 2.1. Setup and Measurement Campaign

The test areas were set up at two different locations in Slovenia, the first one near Stara Vrhnika for the vertical test, while the second was used for horizontal tests and was set up for previous tests in Črnotiče. The main reasons for these locations were that they are located in a remote area where the impact of jamming on users is minimal. In addition, there is almost no traffic at the sites, and the location in Črnotiče allowed outdoor observations because there are no elevated obstacles near the location of the GNSS receivers that would disturb the GNSS signal reception. In addition, the Stara Vrhnika site has a 22-metre-high wooden observation tower, which enabled vertical jamming tests to be performed. Since jammers are illegal to use in Slovenia, the campaign was authorised by the Agency for Communication Networks and Services of the Republic of Slovenia (AKOS).

**Figure 1:**
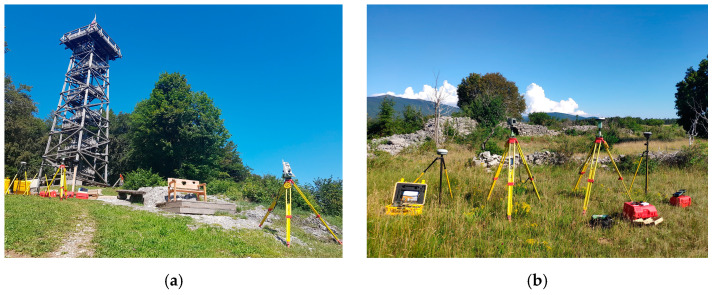
Testing sites in Slovenia: (**a**) near Stara Vrhnika and (**b**) near the village of Črnotiče.

The experiments were conducted on the 202nd day of 2020 (20 July 2020). At the Stara Vrhnika site, the observations were carried out from about 7:15 to 8:45 UTC with a jamming time of 8:15 to 8:40 UTC. At the Črnotiče site, the observations lasted from 13:45 to 15:20 UTC with a jamming time from 14:30 to 14:59 UTC. In the experiments, GNSS receivers from various manufacturers, namely Trimble Inc. (Sunnyvale, CA, United States), Javad GNSS Inc. (San Jose, CA, USA), Leica Geosystems AG (Heerbrugg, Switzerland), ArduSimple (Lleida, Spain), and u-blox (Thalwil, Switzerland) were used, specifically: one Trimble R8S receiver (antenna type: TRMR8S NONE), one Trimble R10 receiver (antenna type: TRMR10 NONE), one Javad Triumph-VS receiver (antenna type: JAVTRIUMPH_VS NONE), three Javad Triumph-LSA receivers (antenna type: JAVTRIUMPH_LSA NONE), three Leica GS15 receivers (antenna type: LEIGS15 NONE), three Leica GS18T receivers (antenna type: LEIGS18 NONE), and three u-blox ZED-F9P modules with ANN-MB00 multi-band GNSS antennas [44,45] (see Figure 1). GNSS receivers were placed close together on tripods and tribrachs. Of these, the Trimble R8S and Javad Triumph-VS were configured for GPS and GLONASS reception, while the others also allowed Galileo observations. Javad Triumph-LSA, Leica GS18T, and u-blox ZED-F9P additionally allowed the reception of BeiDou.

**Figure 2:**
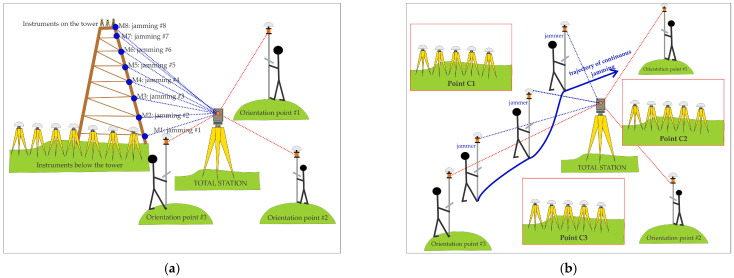
Combined use of global navigation satellite systems (GNSS) and terrestrial positioning system (TPS) (**a**) vertical jamming at Stara Vrhnika; (**b**) horizontal experiments at Črnotiče.

To precisely locate the position of the jammer during the jamming process, Leica SmartPole equipment consisting of a Leica GS18T GNSS receiver, a Leica TS16 total station, and a 360° prism was used. The station setup was performed using a resection method before jamming. Orientation points were determined using the GNSS RTK (real-time kinematic) method in the Slovenian realisation of the ETRS89 coordinate system, D96-17/TM. Figure 2 shows the station setup and further determination of the positions of the jammer for each location. During jamming, the position of the jammer was determined by using a terrestrial positioning system (TPS).

A commercially available in-car jammer was used for the experiments. With reference to Borio et al. [46], it was an unmarked jammer L1/E1 of the subminiature version A (SMA), without data from a manufacturer, powered by a battery; its external antenna with an omnidirectional radiation pattern was connected through an SMA connector, which emitted a single saw-tooth chirp signal, according to [32,35,47], belonging to the group of class II. According to their tests, the output of the jammer has a period of 10 μs, while according to the test carried out for this study, the device increases the noise power by up to 50 dB in a frequency band of 1570 ± 20 MHz. Following the STRIKE3 (Standardisation of GNSS Threat reporting and Receiver testing through International Knowledge Exchange, Experimentation and Exploitation) attempt at standardised threat reporting of jamming events [48], the effect of this particular jammer should be described as a type B interfering event, which exerts a 10 dB decrease of C/N_0_ that lasts longer than 5 s [49].

### 2.2. Vertical Jamming Performance

The vertical jamming experiments followed a pre-planned library of scenarios consisting of setting up the instruments under and on the tower (Table 1), determining the position of the jammer, and determining the static and kinematic jamming mode timings.

The jammer was initially kept static for one minute at different locations on the tower, which ranged about 2–4 m apart (Figure 2). At each location, which was determined by TPS and indicated by M1–M8 (Table 2), static jamming was performed for about one minute. The jammer was turned off when transferred between the two consecutive locations. When reaching the top, a kinematic test was performed after a while (from 8:36:01 to 8:39:29 UTC), during which the operator went down the steps of the wooden tower at a constant speed.

**Figure 3:**
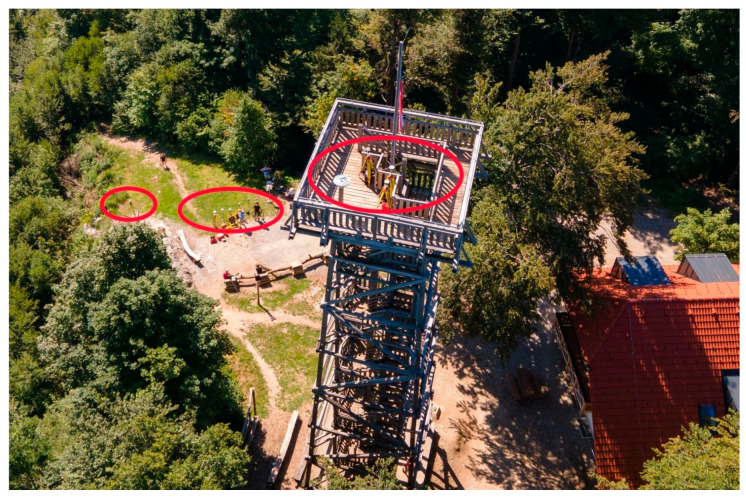
Vertical profile of jamming at the Stara Vrhnika location, top–down view, acquired by a Mavic Air 2, DJI drone. The upper metallic fence structure and flagpole are grounded.

In this scenario, jamming was performed for three minutes intermittently. The Leica GS15, Leica GS18T, Javad Triumph-LSA, and u-blox ZED-F9P were at the top of the tower, while others were mounted below the tower, namely: Javad Triumph-VS, Javad Triumph-LSA, Leica GS15, Leica GS18T, Trimble R8S, Trimble R10, and another u-blox ZED-F9P. Figure 3 shows the positions of the receivers at the top and bottom of the tower, while the start/end times of the jammings and the positions of the jammer are listed in Table 2.

The horizontal distance between the jammer and the GNSS receivers was about 25 m for the receivers below the wooden tower and about 5 m for the receivers on top of the tower. In the vertical direction, the height differences decreased/increased from 26/0 m to ±2–4 m for each individual location of the jammer relative to the receivers at the top and below the tower.

### 2.3. Horizontal Jamming Performance

**Figure 4:**
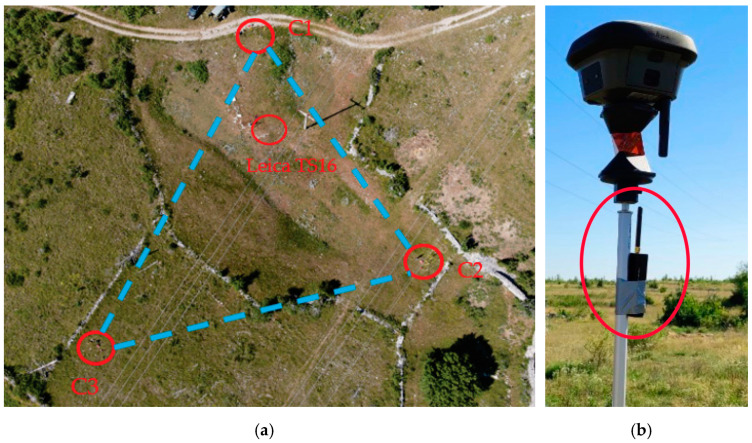
Horizontal profile of jamming at the Črnotiče location (**a**) a top–down view, acquired by a Mavic Air 2, DJI drone; (**b**) the jammer was mounted on a telescopic pole below the 360° prism.

Horizontal jamming tests were carried out at Črnotiče. First, the TPS set up with the resection was performed (see Figure 4). At three different locations, labelled C1, C2, and C3 (Figure 4, Table 3), the receivers were placed so that each location contained at least one receiver from the same manufacturer, namely, location C1, Javad Triumph-LSA, Javad Triumph-VS, Leica GS15, Leica GS18T, and u-blox ZED-F9P; location C2, Javad Triumph-LSA, Leica GS15, Leica GS18T, Trimble R10, and u-blox ZED-F9P; location C3 Javad Triumph-LSA, Leica GS15, Leica GS18T, Trimble R8S, and u-blox ZED-F9P. Each of the receivers performed static observations at a time rate of 1 s.

**Figure 5:**
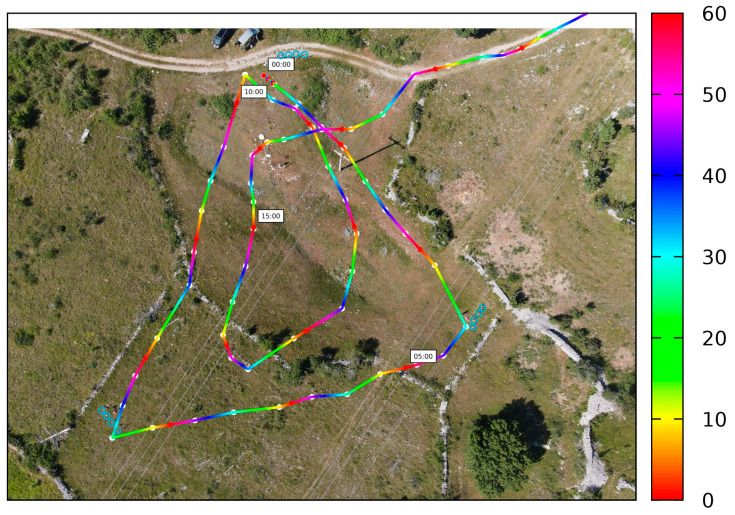
The trajectory of the jammer, defined by TPS, during the kinematic session. The hue of the trajectory represents the time in seconds within a minute. The white dots represent the points at which prism measurements took place.

The horizontal distances between the sites were 74, 82, and 62 m, for $\bar{C1C2}$, $\bar{C1C3}$, and $\bar{C1C3}$ respectively. Continuous kinematic jamming was performed, whereby the operator carried a telescopic pole with a 360° prism on which the jammer was mounted (Figure 4). The trajectory of the jammer was in the inner triangular area, but in some cases also outside. The positions of the jammer were determined every 3 to 5 s, from which a continuous trajectory of the jamming was acquired. Figure 5 presents the trajectory of the jammer, which is defined by TPS, during the kinematic session. The hue of the trajectory represents the time in seconds within a minute. The start of each minute is denoted by a small red dot. There is a label on every five minutes to make reading easier. The white dots represent the points at which the prism measurements occurred. The other points are interpolated.

## 3. Observation Models used for Jamming Detection

Since the double-differencing (DD) of observations effectively eliminates orbital and atmospheric delay errors, it is a widely used positioning technique, in particular because of the added value of the RTK method, which allows direct coordinate acquisition in the national coordinate systems. Signal-to-noise (SNR) values could be used to monitor signal quality. Since interference affects SNR values, the deterioration of positioning quality in the event of float or false fixed ambiguities can be explained by a sudden drop in SNR values. The following describes a basic observation model for the relative positioning approach, the SNR-dependent model, and its application.

### 3.1. DD Observation Model

In DD, the baseline vector must be resolved using the linear model of the observations. Neglecting distortions between the different navigation systems, DD follows the simplified form for the residuals in the code pseudo-range *P* and carrier-phase φ [50]:

(1) $$\begin{array}{l} \nabla ∆v_{P}=\left(\nabla ∆\rho +\nabla ∆M_{P}++\nabla ∆\varepsilon_{P}\right)-\nabla ∆P\\ \nabla ∆v_{\varphi }=\left(\nabla ∆\rho +\nabla ∆N+\nabla ∆M_{\varphi }+\nabla ∆\varepsilon_{\varphi }\right)-\nabla ∆\varphi \end{array}$$

with ∇∆ as DD operator. ρ stands for the distance between satellites and receivers, N is the carrier-phase ambiguity, which should be resolved as an integer, M and ε denote multipath and noise, which are different for code and carrier-phase observations. The model of the variances of the observations is defined according to the elevation angle *E* of the satellite and arbitrarily chosen coefficients a and b, namely [51]

(2) $$\sigma^{2}=a^{2}+\frac{b^{2}}{sin^{2}\left(E\right)}.$$

A detailed description of DD processing with an emphasis on explaining the elimination of impacts on observations can be found in [50].

### 3.2. Signal Strength from GNSS Receivers

The exact information about signal-to noise (SNR) values determination from GNSS receivers of different manufacturers is not always available. Some of the manufacturers give direct definitions of SNR, while the others retain the detailed description for themselves. The fact is that manufacturers can change the approach through the firmware improvement for the particular generation of the receivers. SNR values come for the signal-to-noise counts (SNC) or arbitrary mystery units (AMUs) in the receivers and are scaled to match a measurement over a 1 kHz bandwidth [52]:

(3) $$\mathrm{SNC}\;=S/\sigma_{N},$$

where *S* stands for the amplitude and $\sigma_{N}$ stands for the noise amplitude. The 1 kHz results from the fact that early receivers were integrated for 1 millisecond, resulting in an effective bandwidth of 1 kHz. Furthermore, SNR values are expressed as a power ratio on a logarithmic scale instead of an amplitude ratio on a linear scale. SNR, expressed in the 1 kHz bandwidth, i.e., 1 dB, follows the equation [52]:

(4) $$\mathrm{SNR}\left(\mathrm{dB}\right)=10\cdot log\left({\mathrm{SNC}}^{2}/2\right),$$

or in its simplifications, namely:

(5) $$\begin{array}{c} \mathrm{SNR}\left(\mathrm{dB}\right)=10\cdot log\left({\mathrm{SNC}}^{2}\right)-3\;\mathrm{dB},\\ \mathrm{SNR}\left(\mathrm{dB}\right)=20\cdot log\left(\mathrm{SNC}\right)-3\;\mathrm{dB}. \end{array}$$

SNR expresses the amount by which a signal level exceeds its noise in decibels. SNR values are acquired for each of the frequencies and for each of the satellites, so it is obvious they are elevation dependent.

A more technically precise measurement for GNSS signal strength is C/N0. Some of the receivers have the ability to display such values, but they are determined from directly measured SNC. In the NMEA-0183 GSV sentences, namely $GPGSV and $GLGSV, C/N_0_ values for the GPS and GLONASS satellites are displayed. The C/N_0_ is identified as the carrier power divided by the noise power spectral density per unit bandwidth. It is expressed as follows [53]:

(6) $$C/N_{0}=C-\left(N-\mathrm{BW}\right)=C-N_{0}=\mathrm{SNR}-\mathrm{BW}.$$

where C represents the carrier power in dBm or dBW; N and $N_{0}$ represent the noise power and noise, respectively; and BW is the bandwidth of the observation, which is usually the noise equivalent bandwidth of the last filter stage in the receiver (≈4 MHz, which corresponds to 66 dB for L1 C/A code receiver). C/N_0_ is the SNR (usually in dB) in a 1 Hz bandwidth power density. That bandwidth is 1000 times less than the “standard”, which implies a 30-dB change in dB-power units:

(7) $$C/N_{0}\left(\mathrm{dBHz}\right)=30\;\mathrm{dBHz}+10\cdot log\left({\mathrm{SNC}}^{2}/2\right)$$

or by simplification according to Equation (5)

(8) $$C/N_{0}\left(\mathrm{dBHz}\right)=27\;\mathrm{dBHz}\;+\;10\cdot log\left(\mathrm{SNC}\right).$$

All the above expressions of SNR and C/N0 are approximate but nonetheless useful for expressing weak and strong signals. For example, if SNC is 10, SNR is 17 dB, and C/N_0_ is 47 dBHz, which is a strong signal. A weak signal is equal to SNC 3 and corresponds to the 6.5 dB and 36.5 dBHz for SNR and C/N_0_. C/N_0_ is an important measure that can be used to determine the lock condition of the carrier and tracking loops and to control the channel scheduling [27].

## 4. Data Processing and Evaluation

### 4.1. Carrier-Phase Observation Processing

To determine the positions of the receivers, a relative positioning with a virtual reference station (VRS) near the study area was used as a reference. The VRS was obtained from the Slovenian SIGNAL network of continuously operating reference stations. VRS enabled processing of the shortest baseline, the maximum number of usable constellations, and there were no issues with GLONASS hardware bias caused by the use of different receivers at the end of a baseline. GNSS observations were processed using the RTKLIB software (demo5_b33e) [54,55] using all available navigation constellations and broadcast ephemerides. The ultimate goal of observation processing was to achieve a variation of the solution position over time, so coordinates were acquired for each particular epoch, which provided an insight into the performance of the positioning, especially since the observations were collected during jamming. Since the analyses were performed in post-processing, a combined mode with continuous ambiguity resolution (algorithm LAMBDA [56]) was used instead of the forward mode with activated fix-and-hold. The additional run-through (forward and backward) made it possible to avoid situations in which a fixed solution had to be found without the risk of committing to an incorrect fix that fix-and-hold could cause.

The evaluation approach of jamming comprises two aspects, namely the determination of sudden changes in SNRs, which influence the position quality during or after jamming. First, static GNSS observations at 1 Hz for at least one hour were performed to determine the reliable positions of the receivers, which were then used to determine position errors from jammed observations.

### 4.2. Evaluation of C/N0

**Figure 6:**
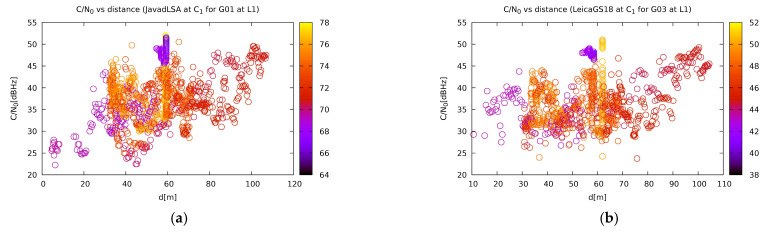

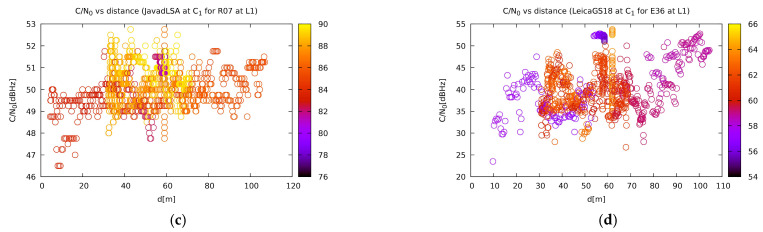
Carrier-to-noise ratio for various satellites and receivers for the horizontal jamming scenario, namely: (**a**) signal for the GPS satellite G01 from Javad Triumph-LSA at site C1; (**b**) signal for the GPS satellite G03 for Leica GS18T at site C1; (**c**) signal for the GLONASS satellite R07 from Javad Triumph-LSA at site C1; (**d**) signal for the Galileo satellite E36 from Leica GS18T at site C1. The colour of the point represents the elevation of the respective satellite. The other plots are available on [57].

However, due to the jamming, the observations were either affected by reduced availability or suffered from ambiguity determination problems, which further affected the positioning accuracy. The proximity of a jammer caused signal losses but not always for all the satellites. Some of the receivers were able to collect observations for GLONASS, while GPS and Galileo had failed. According to expectations, the receivers were more affected when the jammer was close to them. Figure 6 shows some typical carrier-to-noise ratio (C/N_0_) dependencies over the distance of the jammer for various satellites. As expected, the affected GPS and Galileo satellites exhibit a weak yet pronounced dependency. Sometimes, the effect of the jammer approaching can be balanced (or hypothetically even exceeded in the case of a really slow jammer motion) with the increasing elevation of a particular satellite. On the other hand, the GLONASS satellites operate outside the affected frequency band. Their C/N_0_ is almost independent of the vicinity of the jammer, except for a very near region. As seen from Figure 6, signals from GPS and Galileo satellites show a weak correlation between the distance of the jammer and C/N_0_. GLONASS satellites, on the other hand, only become affected in close vicinity when using an L1/E1 chirp jammer.

### 4.3. Positional Quality Assessment

In addition, the study was conducted on the resolution of ambiguities. First, the percentage of fixed and float ambiguities and of cases without resolutions was determined. Based on the awareness that situations with false fixed solutions could occur because of jamming, the same algorithm as in the authors’ previous research [2] was used to estimate the distance between the actual and the measured position. In this case, the first step was to determine the actual position from all observations: φ-latitude λ-longitude and ellipsoidal height (h). Since some of them were jammed and possibly incorrectly fixed, it was not a good idea to simply calculate the mean value of all observations. Based on the use of weighted coefficients, the mean value was instead calculated using:

(9) $$\bar{a}=\frac{1}{N}\overset{N}{\underset{n=1}{\sum }}w_{n}a_{n},$$

where a is any of the position components (φ, λ or h), $w_{n}$ is the weighted function, and *N* is the number of samples. The variance could be obtained in a similar way:

(10) $$\sigma_{a}^{2}=\frac{1}{N-1}\overset{N}{\underset{n=1}{\sum }}w_{n}{\left(a_{n}-\bar{a}\right)}^{2}.$$

Ideally, the weighting function should be selected so that it is 1 for non-jammed samples and 0 for others. The algorithm made it possible to determine the presence of jamming independently of the already known times of jamming, which was particularly advantageous for kinematic jamming scenarios. For this purpose, all $w_{n}$ were initially set to 1 to get a rough estimate of $\bar{a}$ and $\sigma_{a}$ for a further iterative procedure to define $w_{n}$. In the next step, all samples that had at least one of the three quantities from the region $3\sigma_{a}$ were considered jammed, and $w_{n}$ was set to 0 for them. The new weighting function improved the accuracy of $\bar{a}$ and $\sigma_{a}$. Then, the procedure was repeated until no change in $w_{n}$ was detected. This was usually done in five to six steps. After the mean values were obtained, the horizontal position deviations for each sample point *n* could be defined as:

(11) $$\delta Hz_{n}=\left(R+h_{n}\right)\cdot \sqrt{{\left[\left(\varphi_{n}-\bar{\varphi }\right)\cos\left(\lambda_{n}\right)\right]}^{2}+{\left(\lambda_{n}-\bar{\lambda }\right)}^{2}}$$

and where *R* stands for the mean radius of the ellipsoid at the point ($\varphi_{n},\lambda_{n},\;h_{n}$).

## 5. Results and Discussion

The results of both the vertical and horizontal jamming tests showed that the jammer affected the measurements of the specific receivers differently. The performance of the receivers was evaluated according to their ability to calculate their position and the deviation of the solution from the exact value. In addition, the effect on the C/N_0_ of the satellite was calculated, showing reception of the receivers of GPS, GLONASS, Galileo, and BeiDou, if possible. Interestingly, GLONASS reception in the receivers was affected by the presence of the jammer, although the jammer used did not significantly interfere with the frequency band used by the GLONASS satellites.

### 5.1. Vertical Jamming Profile

For the vertical jamming scenario, it was intended that receivers would be positioned on the top of the tower, wherein no significant influence from the jammer was expected due to the antennae radiation pattern of receivers. Non-intentionally, a grounding conductive low-impedance structure apparently transmitted surface electromagnetic waves as interference and caused weak errors, which occurred when the jammer was switched on. It appears that the receivers most affected in the vertical jamming scenario were Javad Triumph-LSA and u-blox ZED-F9P, which are located below the tower and offered no (or almost no) fixed solutions. During jamming, positioning of the receivers below the tower failed to a greater extent or remained in float ambiguity resolution mode. This was the case for all types of receivers located at the bottom of the tower except the Leica GS18T. The response of this receiver was the same regardless of the location (see Table 4 and Table 5).

**Figure 7:**
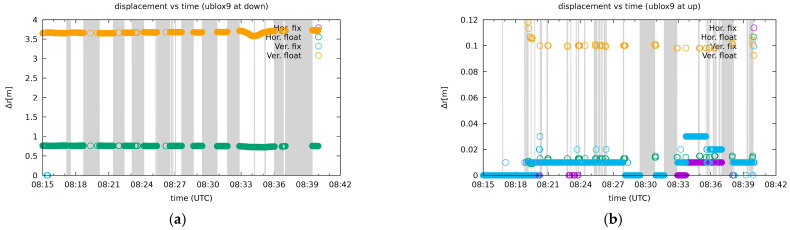
Positioning situation in the vertical jamming scenario for the u-blox ZED-F9P receivers. (**a**) Situation for the receiver below the tower, (**b**) situation for the receiver on the tower. Shaded regions indicate the absence of any solution. The other plots are available on [57].

However, in the overall solution, the receiver most affected by the vertical jamming scenario was the Javad Triumph-LSA. The u-blox ZED-F9P and Javad Triumph-VS receivers performed slightly better, albeit still poorly. In the case of the receiver at the top of the tower, the jamming could be detected by situations with some float solutions for situations where the jammer was below the receiver or without a solution that occurred when the jammer was at the same level or above the receiver. However, the receivers below the tower suffered much more, as shown in the left in Figure 7. This could be attributed to the receiving antenna that is probably designed for better reception of the signals in the upward direction.

**Figure 8:**
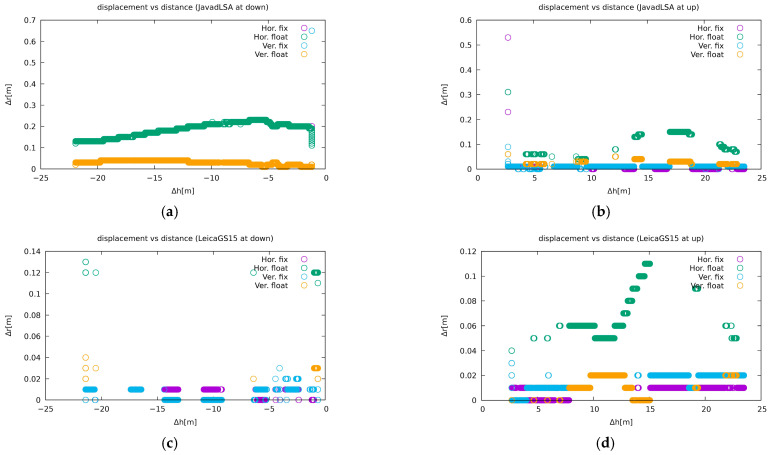
Solution calculations of a particular receiver based on the jammer position for Javad Triumph-LSA at (**a**) the bottom of the tower and (**b**) on the tower; Leica GS15 at (**c**) the bottom of the tower and (**d**) on the tower. A negative value means the jammer is above the receiver and below when positive. The other plots are available on [57].

The jammer caused signal losses, and in available positioning situations between successive one-minute static jamming, the receiver was unable to resolve the ambiguities as fixed and remained in the float solution mode. The Javad Triumph-LSA below the tower beside a low proportion of phase fix solutions (or any solution at all) yielded a few apparently wrong solutions that it claimed to be phase fixed. Results for two receivers are presented in Figure 8: a poorly performing Javad Triumph-LSA (upper plots) and an example of a good performing Leica GS15 (lower plots). The left plots represent the receivers below the tower, while the right ones denote the receivers on the top.

The first plot in Figure 8 shows carrier-phase fix solutions (a single point as they are close to each other) with a displacement of up to 0.7 m in the vertical direction and up to 0.2 m in the horizontal. The same figure also shows the Javad Triumph-LSA receiver on the top of the tower, where it performs much better, although there were a few phase fix solutions deviating up to 0.5 m in the jammer proximity. The same figure also shows an example of a receiver that performed better: Leica GS15. The receiver at the top of the tower provided more solutions compared to the one below the tower. However, those solutions were mostly the carrier-phase floating type.

**Figure 9:**
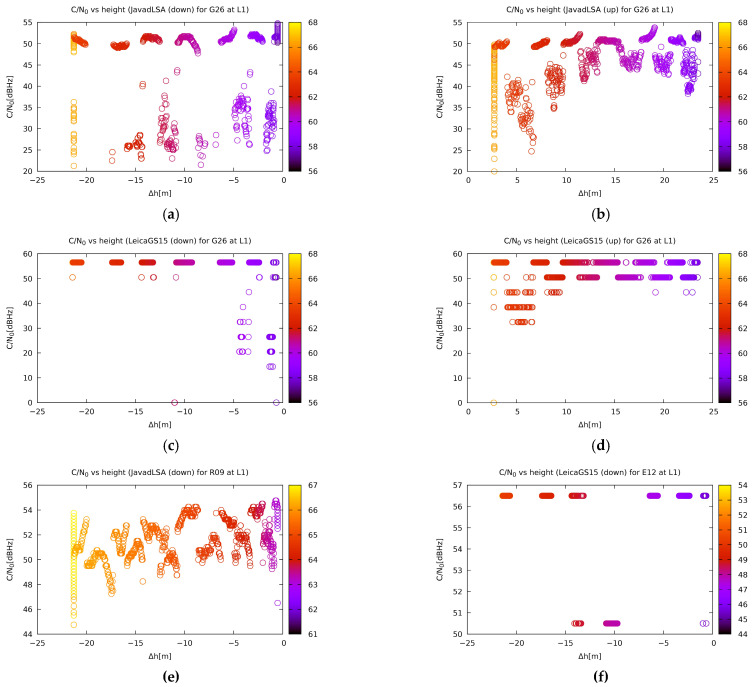
Carrier-to-noise-zero (C/N0) dependence on the relative vertical distance of the jammer for some receivers and satellites: the signal for the GPS satellite G26 from Javad Triumph-LSA at (**a**) the bottom of tower and (**b**) on the tower; for Leica GS15 (**c**) at the bottom of the tower and (**d**) on the tower; (**e**) GLONASS satellite R09 from Javad Triumph-LSA from down and (**f**) signal for Galileo satellite E12 from Leica GS15 from down. Colour of the points represents the satellite elevation. The other plots are available on [57].

**Figure 10:**
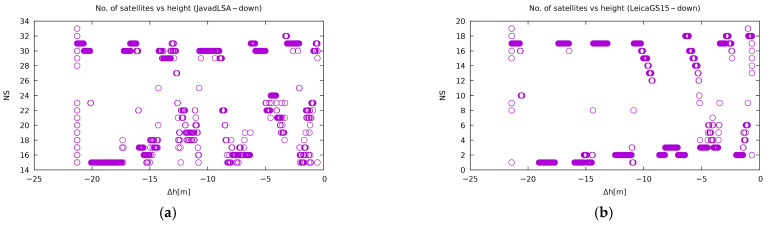
Number of satellites (NS) with respect to the relative vertical distance of the jammer for: (**a**) Javad Triumph-LSA and (**b**) Leica GS15. The other plots are available on [57].

The next figure (Figure 9) shows the same two receivers and their reception of the G26 satellite of the GPS constellation at both positions. Again, Leica GS15 had better reception compared to Javad Triumph-LSA, and both receivers receive better at the top. The last two plots represent the reception of the R09 of the GLONASS constellation on the Javad Triumph-LSA and E12 of Galileo on Leica GS15. The GLONASS satellites obviously have a lower C/N_0_ drop since they are out of the jamming frequency band. On the other hand, the Galileo satellites show an instantaneous drop of their signal once jammed. As a curiosity, Figure 10 shows the number of satellites used to compute the position. It is interesting that Javad Triumph-LSA uses more satellites than Leica GS15. Furthermore, Leica GS15 has a particularly big proportion of acquisition of less than the theoretical minimum of four satellites. Nevertheless, it outperforms Javad Triumph-LSA.

### 5.2. Horizontal Jamming Profile

The horizontal jamming scenario followed the kinematic mode of the jammer location. Comparing the response behaviour of different receiver types at the same location, namely C1, C2, and C3 (Table 6, Table 7 and Table 8), first, it becomes clear that the geodetic receivers performed much better than the u-blox ZED-F9P low-cost receivers, although Javad Triumph-LSA did not perform much better by means of position precision, as will be shown shortly. In all situations, especially at the site C1, the u-blox ZED-F9P type suffered from a situation whereby no one solution was much better than the others (33.6% compared to 10% for the remaining receivers at the C1 location), while among all the possible solutions at all locations, the float mode of ambiguity resolution prevailed (Table 6). In this scenario, it is obvious that geodetic receivers perform better, because they are equipped with much more effective approaches against interference that affect the resolution of ambiguities or the possibilities of an incorrect carrier-phase fix. However, low-cost receivers near continuously operating reference stations (CORS) could be used for the early detection of interference.

**Figure 11:**
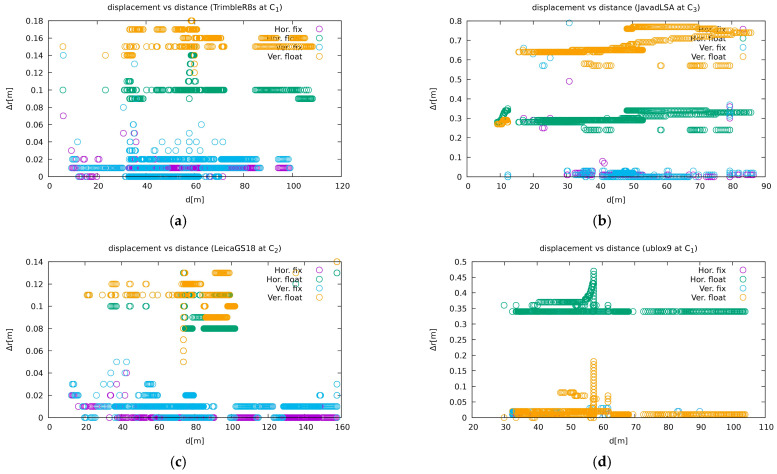
Examples of the precision dependency of receivers on the jammer vicinity, namely: (**a**) Trimble R8S at site C1; (**b**) Javad Triumph-LSA at site C3; (**c**) Leica GS18T at site C2; and (**d**) u-blox ZED-F9P. The other plots are available on [57].

At site C1, Trimble R8S showed the best performance in available positioning (9% versus 10% to 16% for other geodetic receivers) and also in ambiguity fixing (59% of all available solutions); see Table 6. The other receivers at site C1 performed similarly well but slightly worse than the Trimble R8S. Additionally, by means of precision, Trimble R8S superseded the others (first plot in Figure 11).

As can be seen in Table 7, the performance of the Trimble R10 receiver at site C2 was successful with the available solution (more than 90%), but only 5% of them were able to achieve the best quality, i.e., integer ambiguity resolution. The remaining receivers, if u-blox ZED-F9P is excluded, achieved phase fixes of 46, 48, and 72% respectively for the Leica GS18T, Javad Triumph-LSA, and Leica GS15.

At site C3, Javad Triumph-LSA was most successful in available positioning (22% vs. 30% or more without solutions), but of all the possible solutions, the Leica GS15 was most successful in carrier-phase fixing (see Table 8). However, a large proportion of Javad Triumph-LSA’s solutions were not accurate (second plot in Figure 11), some of them even being labelled as carrier-phase fixed. The Leica GS18T performed similarly but slightly worse than the Leica GS15. The Javad Triumph-VS did not perform well, its performance was comparable to the u-blox ZED-F9P receiver.

When comparing the same receiver type at different locations (Table 6, Table 7 and Table 8), the Leica GS18T was most resistant to interference at site C1, while the Leica GS15 was most resistant at site C2, and their effectiveness was quite similar at site C3. While the Trimble R8S at site C1 was comparable to the other receivers except u-blox ZED-F9P, the Trimble R10 at site C2 was not as successful. Its performance was even worse than that of the u-blox ZED-F9P receiver.

**Figure 12:**
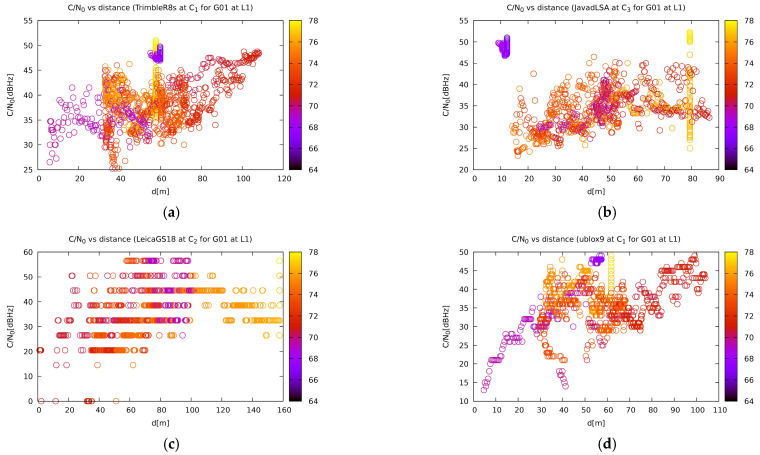
Reception of the G01 satellite of the GPS system by various receivers: (**a**) Trimble R8S at site C1; (**b**) Javad Triumph-LSA at site C3; (**c**) Leica GS18T at site C2; (**d**) u-blox ZED-F9P at site C1. The other plots are available on [57].

In terms of satellite reception, as previously mentioned, the correlation between the jammer distance and the carrier-to-noise ratio is very weak. However, it can be noted that the receivers that have better performance in a particular measurement have a more noticeable dependency (Figure 12). The only exceptions are the u-blox based ZED-F9P receivers, which have a very striking dependency despite performing poorly. The authors suspect that this could be attributed to a less sophisticated algorithm that does not take the previous measurements much into account and the position calculation relies more on immediate signal acquisition. Although no direct proof can be given for this statement, there are some indications that point in this direction. It has been seen in previous experiments [2] that geodetic instruments report the correct position in some short time just after the jamming started and need some time to recover after it. As previously discussed, the GPS and Galileo satellites are affected in a similar way, while the GLONASS reception is only affected in close proximity to the jammer.

**Figure 13:**
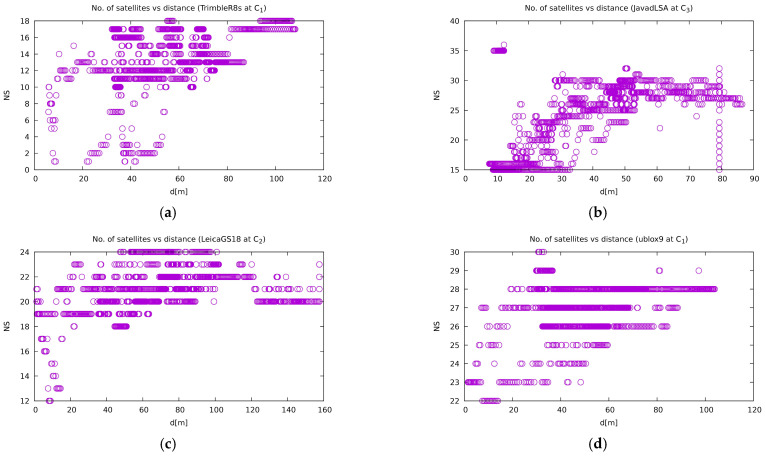
Number of satellites used in the position calculation for the same set of receivers as in Figure 11 and Figure 12. (**a**) Trimble R8S at site C1; (**b**) Javad Triumph-LSA at site C3; (**c**) Leica GS18T at site C2; (**d**) u-blox ZED-F9P at site C1. The other plots are available on [57].

The number of satellites used does not differ much from the vertical jamming experiment. Once again, Figure 13 shows that the use of more satellites in a position calculation does not mean better precision. However, the correlation between the number of satellites and the distance between a receiver and jammer is very prominent.

## 6. Conclusions

This paper presents the evaluation of the performance of geodetic and low-cost GNSS receivers under chirp L1/E1 jamming, which was performed vertically and horizontally. With the knowledge of the competent authorities, jamming experiments were performed on the same day—20 July 2020—at specific times and at two different locations in Slovenia. The jammer was placed at the first vertical and the second horizontal at different locations, and observations from the GNSS receivers were acquired simultaneously in order to obtain information about the respective weak point of each type of receiver depending on the position of the jammer. The position quality and the potential for detection of the displacement of geodetic and low-cost GNSS receivers in relation to the knowledge about the presence of the jammer was further investigated. The results obtained in this study lead to the following conclusions:

- In situations where the position of a jammer is above a GNSS receiver, the susceptibility and poor performance of the receiver are much more pronounced than in situations where the position of a jammer is below the same type of the receiver. The statement is even more pronounced in the case of low-cost receivers, which obviously do not contain any interference mitigation to the same extent as geodetic receivers;
- The latest generation of geodetic receivers, such as the Leica GS18T, responded similarly regardless of whether the jammer was below or above the horizon of the receiver, while other receivers responded better to situations where the jammer was below their horizon;
- Both in the vertical and horizontal experiments, u-blox ZED-F9P suffered the most from situations with jamming. When positioning was possible, it usually remained in float mode;
- While the Trimble R8S at site C1 was comparable to the other receivers except u-blox ZED-F9P, the Trimble R10 at site C2 was not as successful; the reason for this could be the small size and different geometry of the Trimble R10 receiver’s antenna, according to the authors’ current opinion;
- The reception of GLONASS satellites is almost unaffected, except in close proximity (10 m or less) to the jammer, as can be seen from their C/N_0_ values.

However, it should be noted at this point that all the tests were carried out under favourable conditions, without any other sources of disturbances that could constitute an additional source of interference. The authors assume that the results would be worse under other conditions, especially in suburban and urban scenarios. Therefore, it would be useful to conduct further tests in the future, in which several additional factors should be analysed. In further studies, the authors will analyse a wider range of low-cost navigation receivers, including different types of antennas. The receivers should be analysed to determine their performance separately for each antenna and receiver type.

The performance of such experiments and the interpretation of the results is always somewhat limited. There are many factors in the overall workflow that influence the results. Nevertheless, the authors believe that the results and conclusions from their experiments contribute to a better understanding of the effects of problems that occur when using GNSS receivers in the event of jamming. To this end, further work will focus on identifying the reasons for the elimination of false ambiguity fixing and the quality of jammer localisation in different jamming scenarios.

Another direction of research could also be the investigation of the impact of jammers on GNSS reception in some less idealistic environments where reflections and involuntary electromagnetic interferences are present (e.g., urban areas). However, a permission from the authorities to perform such kinds of experiments would probably be impossible to obtain. Despite those limitations, we already thought of performing a simulation of those situations, such as in an abandoned remote village with a lot of geographic obstacles with the presence of some equipment that is known to produce a lot of interference.

## Figures and Tables

**Table 1:** Positions of the receivers at the Stara Vrhnika in the Slovenian realisation of the ETRS89 coordinate system. The receivers were set up under and on the top of the wooden tower. Normal heights (H) were acquired from the ellipsoidal heights (h) by using the SLO_VRP2016/Koper geoid model.

Receivers’ Position	B-Latitude	L-Longitude	h [m]	H [m]
On the tower	45°58′17.6″ N	14°15′06.0″ E	802.9–803.2	756.3–756.6
Below the tower	45°58′17.2″ N	14°15′06.9″ E	777.3–778.9	730.7–732.3

**Table 2:** Locations of the jammer and times of jamming in UTC at the Stara Vrhnika location (the Slovenian realisation of the ETRS89 coordinate system).

Location of Jammer	Start and End Times of Jamming (UTC)	B-Latitude	L-Longitude	h [m]	H [m]
M1	8:17:10–8:17:32	45°58′17.4938″ N	14°15′06.1969″ E	780.904	734.295
M2	8:18:42–8:19:57	45°58′17.5938″ N	14°15′06.0527″ E	783.072	736.463
M3	8:21:24–8:22:24	45°58′17.5442″ N	14°15′06.0827″ E	785.923	739.314
M4	8:23:06–8:24:11	45°58′17.5527″ N	14°15′06.0782″ E	789.905	743.296
M5	8:25:22–8:26:33	45°58′17.5381″ N	14°15′06.0878″ E	793.526	746.917
M6	8:27:36–8:28:42	45°58′17.5397″ N	14°15′06.0872″ E	797.321	750.712
M7	8:29:28–8:30:50	45°58′17.5446″ N	14°15′06.1431″ E	802.871	756.262
M8	8:31:45–8:32:51	45°58′17.5446″ N	14°15′06.1431″ E	803.471	756.862
M8 → M1	8:36:01–08:39:29	kinematic jamming from M8 to M1			

**Table 3:** Locations of the jammer and times of jamming in UTC at the Stara Vrhnika location (the Slovenian realisation of the ETRS89 coordinate system).

Site	B-Latitude	L-Longitude	h [m]	H [m]
C1	45°33′45.8″ N	13°53′43.1″ E	477.1–478.2	431.7–432.9
C2	45°33′45.7″ N	13°53′39.8″ E	478.7–479.3	433.3–434.0
C3	45°33′45.7″ N	13°53′42.0″ E	477.6–479.0	432.2–433.7

**Table 4:** Positioning quality with ambiguity resolution for the vertical profile of jamming for the receivers at the top of the tower at the Stara Vrhnika site.

		Quality of Carrier-Phase Ambiguity Resolution		
Receiver Type	Site	Phase Fix	Phase Float	No Solution
Javad Triumph LSA	Stara Vrhnika, on the top	74.4%	15.6%	10.0%
Leica GS15	Stara Vrhnika, on the top	68.5%	22.2%	9.3%
Leica GS18T	Stara Vrhnika, on the top	88.1%	0.4%	11.5%
u-blox ZED-F9P	Stara Vrhnika, on the top	75.5%	4.8%	19.7%

**Table 5:** Positioning quality with ambiguity resolution for the vertical profile of jamming for the receivers below the tower at the Stara Vrhnika site.

		Quality of Carrier-Phase Ambiguity Resolution		
Receiver Type	Site	Phase Fix	Phase Float	No Solution
Javad Triumph LSA	Stara Vrhnika, below	0.2%	92.4%	7.4%
Javad Triumph-VS	Stara Vrhnika, below	1.5%	85.2%	13.3%
Leica GS15	Stara Vrhnika, below	47.1%	6.9%	46.0%
Leica GS18T	Stara Vrhnika, below	90.9%	7.7%	1.3%
Trimble R8S	Stara Vrhnika, below	53.6%	13.7%	32.7%
Trimble R10	Stara Vrhnika, below	56.7%	12.5%	30.8%
u-blox ZED-F9P	Stara Vrhnika, below	0.3%	53.3%	46.5%

**Table 6:** Positioning quality with ambiguity resolution for the horizontal jamming profile for the receivers at location C1, Črnotiče.

		Quality of Carrier-Phase Ambiguity Resolution		
Receiver Type	Site	Phase Fix	Phase Float	No Solution
Javad Triumph LSA	Črnotiče, C1	49.9%	39.3%	10.8%
Leica GS15	Črnotiče, C1	53.9%	30.3%	15.8%
Leica GS18T	Črnotiče, C1	53.7%	23.9%	12.3%
Trimble R8S	Črnotiče, C1	58.8%	32.1%	9.2%
u-blox ZED-F9P	Črnotiče, C1	6.7%	59.7%	33.6%

**Table 7:** Positioning quality with ambiguity resolution for the horizontal jamming profile for the receivers at location C2, Črnotiče.

		Quality of Carrier-Phase Ambiguity Resolution		
Receiver Type	Site	Phase Fix	Phase Float	No Solution
Javad Triumph LSA	Črnotiče, C2	47.7%	41.3%	10.9%
Leica GS15	Črnotiče, C2	71.8%	17.2%	11.0%
Leica GS18T	Črnotiče, C2	46.3%	38.2%	15.4%
Trimble R10	Črnotiče, C2	5.1 %	38.9%	9.1%
u-blox ZED-F9P	Črnotiče, C2	0.3%	81.7%	18.1%

**Table 8:** Positioning quality with ambiguity resolution for the horizontal jamming profile for the receivers at location C3, Črnotiče.

		Quality of Carrier-Phase Ambiguity Resolution		
Receiver Type	Site	Phase Fix	Phase Float	No Solution
Javad Triumph LSA	Črnotiče, C3	37.6%	39.8%	22.6%
Javad Triumph VS	Črnotiče, C3	15.4%	48.7%	35.9%
Leica GS15	Črnotiče, C3	50.9%	15.7%	33.4%
Leica GS18T	Črnotiče, C3	47.8%	23.3%	28.9%
u-blox ZED-F9P	Črnotiče, C3	18.3%	48.3%	33.4%

## Data Availability

The data that support the findings of this study are openly available at https://gnss.fpp.uni-lj.si/2020-07-20. Additional data can be available on request from the corresponding author.
